# Carbonation and serpentinization of diopsidite in the Altun Mountains, NW China

**DOI:** 10.1038/s41598-022-25612-5

**Published:** 2022-12-09

**Authors:** Dingkui Zhou, Shuyun Cao, Jianhua Liu, Xiaowen Li, Yanlong Dong, Franz Neubauer, Jie Bai, Hu Li

**Affiliations:** 1 State Key Laboratory of Geological Processes and Mineral Resources, School of Earth Sciences, China University of Geosciences, Wuhan, 430074 China; 2 Department of Environment and Biodiversity, Paris-Lodron-University of Salzburg, Hellbrunner Str. 34, 5020 Salzburg, Austria

**Keywords:** Geochemistry, Geology, Mineralogy, Petrology

## Abstract

Mineral carbonation of mafic–ultramafic rocks has been highlighted as a promising way for permanent carbon capture and storage. Carbonatization involves the release of Ca, Mg and Fe from silicate minerals by dissolution and reaction in the aqueous phase to form stable carbonate minerals. Diopside is one of the most abundant mafic minerals in the lithosphere and contributes a portion of Mg and Ca to surface weathering. Here, we present detailed processes of the carbonation-coupled serpentinization of diopsidite from the Yushishan Nb–Ta deposit in the Altun Mountain, northwest China. Diopsidite is the prograde metamorphic product of siliceous dolomitic marble by full decarbonation process. Retrograde serpentinization and carbonation of diopsidite lead to the addition of CO_2_, H_2_O, light rare earth elements and fluid-mobile elements but the loss of SiO_2_. The diopsides are replaced by calcite and chrysotile by mineral alteration to form pseudomorphic textures. Dissolution–precipitation processes significantly affect diopside serpentinization and carbonation. The carbonation of diopside-rich rocks may be suitable for permanent CO_2_ storage.

## Introduction

Mineral carbonation has been considered a safe and promising process enabling essentially permanent carbon capture and storage (CCS)^1–6^. It captures CO_2_ to react with silicate minerals and immobilize CO_2_ in stable carbonate products such as calcite (CaCO_3_), dolomite (Ca_0.5_Mg_0.5_CO_3_), magnesite (MgCO_3_) and siderite (FeCO_3_) over human timescales^1–8^. In nature, large volumes of mafic–ultramafic rocks have been studied to record carbonation processes, especially basaltic rocks, which are rich in calcium, magnesium, iron oxides and highly porous^9–12^ showing very promising potential for carbon storage. Neither carbonation nor serpentinization of diopside-rich diopsidite have ever been described in detail for natural samples, although some experimental studies have explored carbonation processes of diopside^13,14^.

Pyroxene is one of the main rock-forming minerals in the Earth's mantle lithosphere (e.g., peridotite and pyroxenite) and crust (e.g., gabbro, basalt, and diopsidite). Early studies were focused on the dissolution mechanism of pyroxene and suggested selective dissolution^15^, surface chemical reaction^16^ and migration of water to the pyroxene surface^17^. Furthermore, many factors, such as the initial states of minerals^18,19^, pH of the solution^20–22^, temperature^21,23^ and solution composition^22,24^, can influence the dissolution processes of pyroxene. In recent years, it has been gradually realized that pyroxene (especially diopside) is a promising mineral for carbon dioxide sequestration during the carbonation process^13,25^. Experimental studies found that diopside can be altered to talc and serpentine by Ca release during serpentinization of diopside^26,27^. Moreover, serpentinization and chloritization occur in natural clinopyroxenite, which can provide the main Ca source for rodingitization of mafic rocks^28,29^. However, few studies have been performed on pyroxene carbonation and coupling of the process with serpentinization.

It has been suggested that peridotite in seafloors and subduction zones is carbonated and serpentinized^30^. Case studies from the Oman ophiolite show that peridotites react with CO_2_-bearing fluids to form large amounts of serpentine and carbonate^31–33^, and with an estimated rate of natural carbonation of about 10^4^–10^5^ tons CO_2_/yr^31^ and 10^6^–10^7^ kg CO_2_/yr^33^. Residual olivine is surrounded by antigorite and magnesite, and serpentinization and carbonation can occur simultaneously during olivine alteration^34^. During carbonation, peridotite can be converted into ophicarbonate (carbonate-rich serpentinite), soapstone (talc + magnesite + serpentine) and listvenite (quartz + magnesite and/or dolomite + talc)^32,35–38^. Similarly, exposed peridotite can react with seawater (in ancient peridotite-hosted hydrothermal systems) to form serpentine and calcite^39^. Interestingly, recent studies also suggest that serpentinization and carbonation of minerals are closely related to organic synthesis on terrestrial planets^40^.

In this work, we report new insights into the processes of serpentinization and carbonation of diopside in diopsidite from the Yushishan Nb–Ta deposit in the eastern part of the Altun Mountains, northwest China. Mineral assemblages and microstructures show obvious features of mineral alteration. Bulk-rock geochemistry and mineral composition reveal coupled serpentinization and carbonation of diopside. These results also allowed us to probe potential permanent carbon reservoirs over geological timescales.

### Geological setting and sample selection

**Figure 1:**
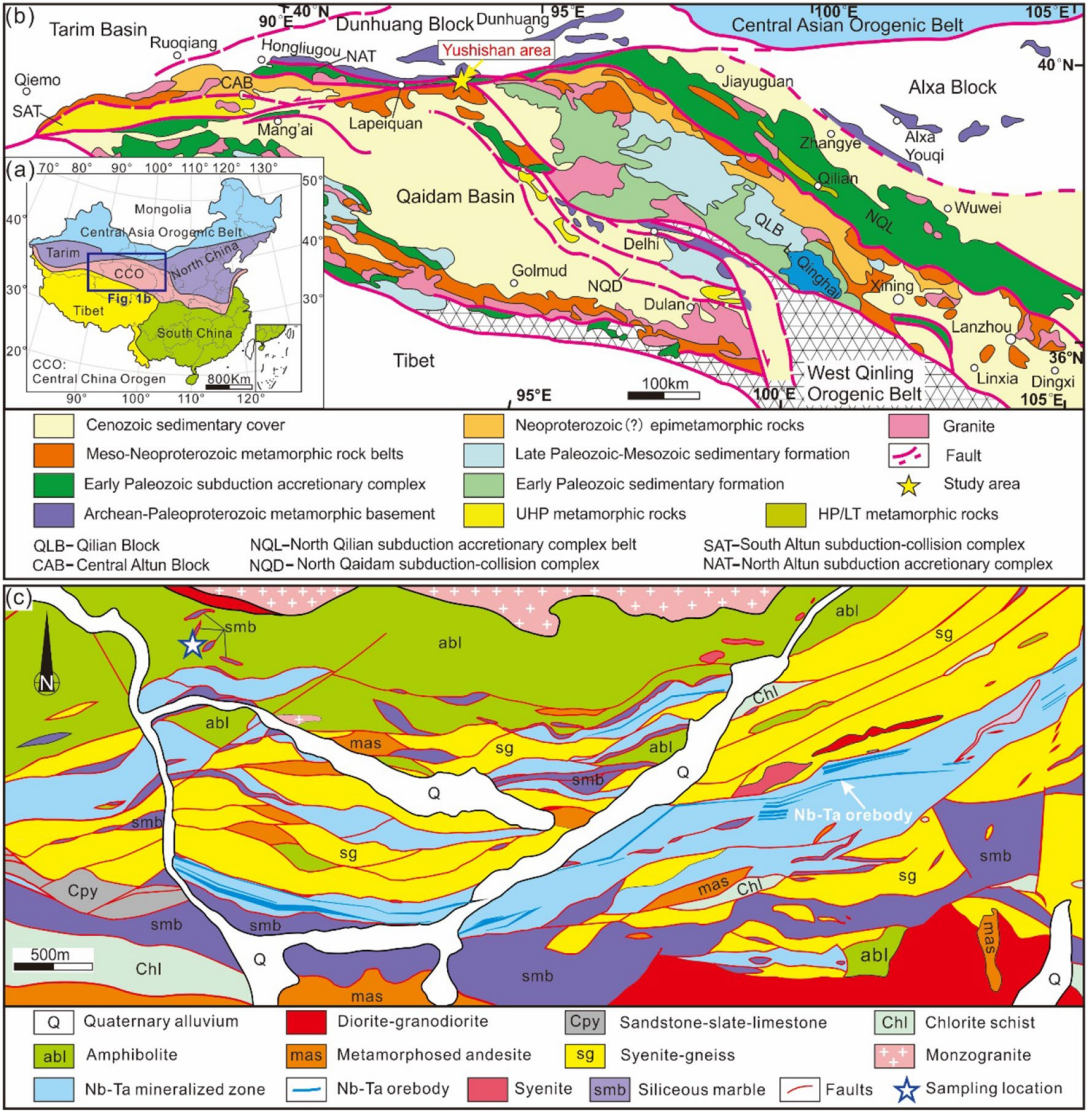
Geological map. (**a**) Simplified geological map of China (modified from Yu et al.^41^). (**b**) Geological sketch map of the Altun–Qilian–Kunlun (AQK) orogenic system in northern Tibet (modified from Zhang et al.^42^). (**c**) Geological map of the Yushishan Nb–Ta deposit (from the Geological Survey of Gansu Province, China). The figure was created using CorelDRAW 2018 software (https://www.coreldraw.com/en/pages/coreldraw-2018/), and the map will not have a copyright dispute.

The Yushishan area in the eastern Altun Mountains is a newly discovered rare metal (Nb, Ta) deposit, which is located at the junction of the Altun Block and Qilian Block that belong to the Central China Orogen^41,42^ (Fig. 1). Exposed lithologies in the Yushishan mainly include the Paleoproterozoic Dakendaban Group, Mesoproterozoic Aoyougou Formation, Neoproterozoic Binggounan Formation, Cambrian–Ordovician Lapeiquan Formation and Carboniferous Yanghugou Formation. The Aoyougou Formation is the host stratum of the deposit, which mainly consists of siliceous marble, amphibolite, chloritic schist, meta-andesite, granitic gneiss, syenite and gneiss (Fig. 1c). The host rocks of the Nb–Ta ore bodies are mainly gneiss and syenite, which are in close contact with siliceous marble. Corundum within syenite and gold-bearing quartz veins have been reported in the deposit^43,44^.

These rare studies suggest that the Yushishan deposits have undergone multiple periods of tectono-magmatism and metamorphism, mainly during Proterozoic and Palaeozoic times^45,46^. The developed migmatite and metamorphic rocks probably experienced pressure granulite-facies metamorphism, although there are no exact P‒T data from previous studies. It has been suggested that the processes of Nb–Ta mineralization were closely related to early Palaeozoic magmatic-metamorphic events^46^. The presence of mineral inclusions with Nb (columbite-(Fe), samarskite-(Y)) in corundum suggests that melting and hydrothermal fluid processes have changed the physico-chemical character and led to Nb and Ta precipitation in high-temperature metamorphic gneiss^44^. Gold-bearing quartz veins are exposed in this area, which are controlled by regional ductile shear zones and brittle faults^43^. The ore-forming fluids are dominated by magmatic-hydrothermal fluids and are characterized as CO_2_-rich.

**Figure 2:**
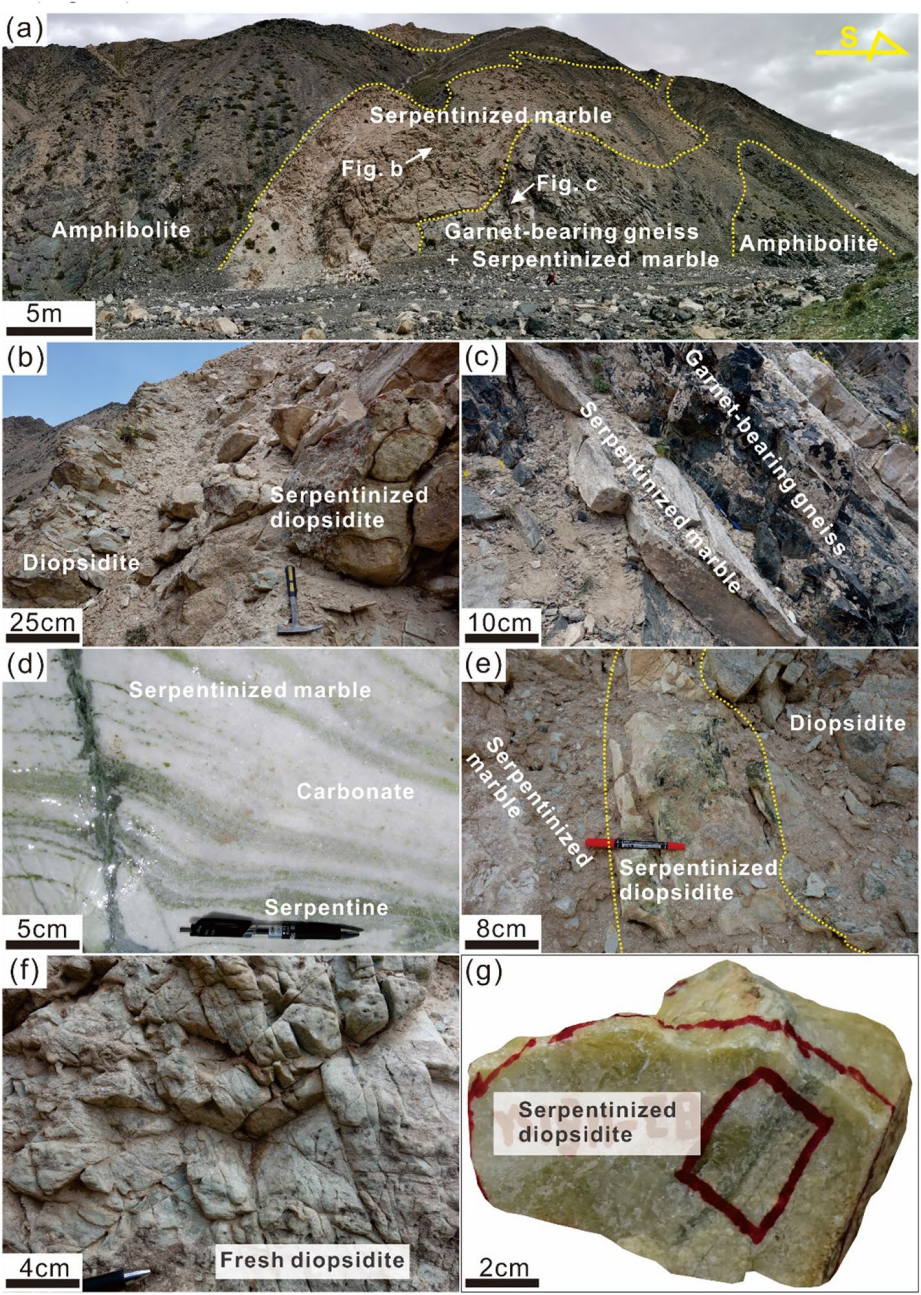
Field photographs of serpentinized diopsidite in the Yushishan deposits. (**a**) Serpentinized marble with amphibolite and interbedded garnet-bearing gneiss. (**b**) Diopsidite associated with serpentinized diopsidite. (**c**) Serpentinized marble interbedded with garnet-bearing gneiss. (**d**) Serpentinized marble comprises carbonate (dolomite or calcite) and serpentine. (**e**) Diopsidite with serpentinized diopsidite and serpentinized marble. Note that there is obvious zonation among them. (**f**) Fresh diopsidite develops joints. (**g**) Hand sample of a serpentinized diopsidite.

The studied diopsidites are exposed in the northwestern part of the Yushishan deposits that occur together with amphibolite, serpentinized marble (such as olivine-bearing marble) and garnet-bearing gneiss (Figs. 1c and 2a). Fresh diopsidite and serpentinized diopsidite can be observed at the same outcrop (Fig. 2b). The serpentinized marble is interbedded with garnet-bearing gneiss (Fig. 2c), which mainly consists of serpentine, dolomite and calcite (Fig. 2d). A few marbles contain forsterite which was locally serpentinized. Moreover, some serpentinized marbles also contain diopside and tremolite. In addition, several serpentinized marbles are composed of calcite and antigorite. It is worth noting that diopsidite is included in serpentinized marbles (Fig. 2e).

## Results

### Microstructures and minerals of fresh and serpentinized diopsidites

**Figure 3:**
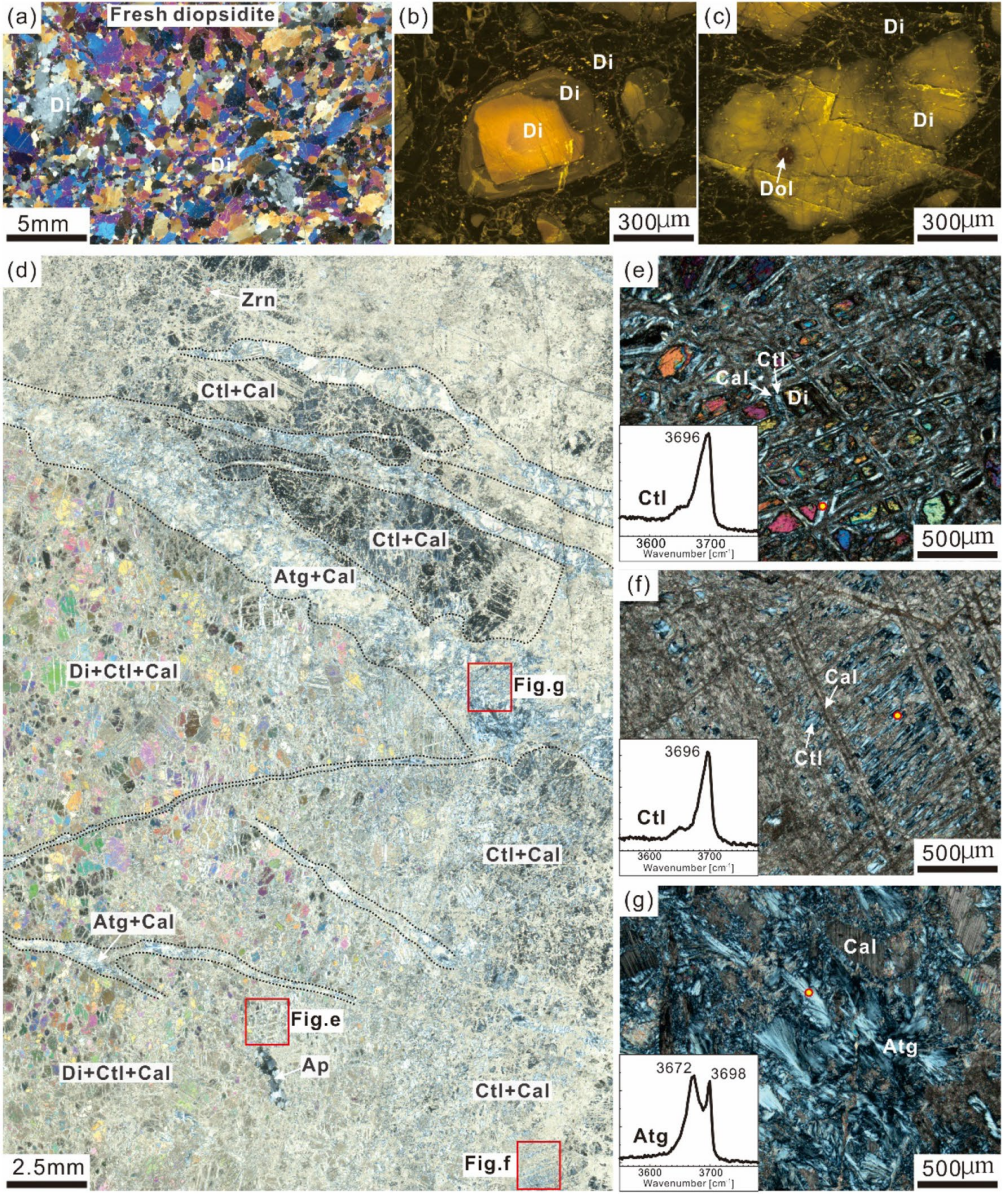
Microstructural features of fresh and serpentinized diopsidites from the Yushishan area. (**a**, **b**, **c**) Fresh diopsidites, (**b**, **c**) cathodoluminescence images, (**d-g**) photomicrographs of serpentinized diopsidite with the Raman peaks of chrysotile and antigorite. (**a**) Microstructure of fresh diopsidite under crossed‐polarized light. (**b**) Diopsides (Di) show the texture of compositional zoning. (**c**) Cathodoluminescence images, diopside including dolomite (Dol). (**d**) Image of scanned full thin section showing heterogeneous serpentinization of diopside (Di) as well as with a late antigorite (Atg) + calcite (Cal) vein, apatite (Ap), zircon (Zrn). (**e**) Serpentinization occurred along the cleavage plane of diopside forming chrysotile (Ctl) + calcite (Cal) + relict diopside. (**f**) Completely serpentinized pseudomorph with chrysotile + calcite. (**g**) Late antigorite (Atg) + calcite vein.

Fresh diopsidite is grayish green (Fig. 2f). It is mainly composed of subhedral to anhedral diopside with varied grain sizes ranging from 10 μm to 10 mm (Fig. 3a). The diopside shows inhomogeneous compositional zones (Fig. 3b). Some small dolomites are included in a few of the diopside grains (Fig. 3c). The serpentinized diopsidite exhibits a variety of colours ranging from yellowish white to yellowish green (Fig. 2e, g). It mainly consists of diopside, calcite and serpentine and the secondary minerals minor zircon and apatite (Fig. 3d–g).

The serpentine includes chrysotile and antigorite, which was identified with optical microscopy and Raman spectroscopy (Fig. 3e–g). Lizardite may also occur, but that could not be determined in this study. Chrysotile shows the characteristic band at 3696 cm^−1^ (Fig. 3e, f), and antigorite is confirmed by the characteristic bands at 3672 cm^−1^ and 3698 cm^−1^ (Fig. 3g). Most diopsides were completely replaced by chrysotile and calcite to form networks of chrysotile and dominant calcite. Some serpentinization occurs along the cleavage planes of diopside, forming chrysotile (Ctl) + calcite (Cal) + residual diopside (Fig. 3e). The diopside relics with anhedral grains are surrounded by chrysotile and calcite. The pseudomorphic replacements of chrysotile and calcite after diopside retain the cleavage in one or two directions of the diopside crystals (Fig. 3d, f). Antigorite together with calcite occur in small veins (Fig. 3d, g).

**Figure 4:**
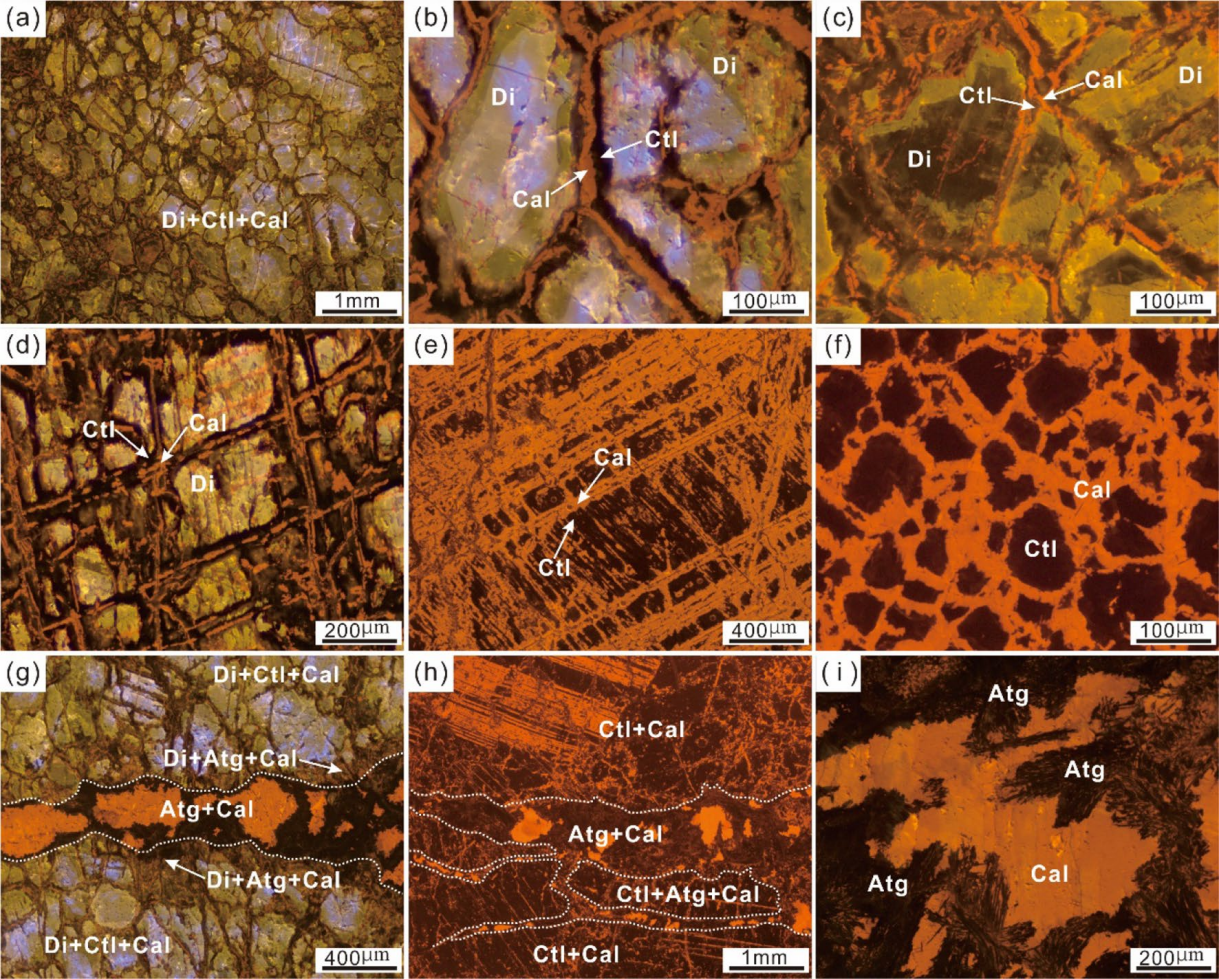
Optical cathodoluminescence (CL) images of serpentinized diopsidite from the Yushishan area. (**a**) Serpentinized diopsides with inhomogeneous cathodoluminescence. (**b**) Serpentinization occurred around a diopside grain (Di) and formed chrysotile (Ctl) + calcite (Cal). (**c**) Chrysotile and calcite formed along the cleavage plane of diopside. (**d**) Relict diopside rimmed by a layer of chrysotile followed by a layer of calcite. (**e**) Complete serpentinization of diopside forming along cleavages. (**f**) Complete serpentinization of diopside forming pseudomorphs of chrysotile + calcite with network fabrics. (**g**) Serpentinized diopsides crosscut by a late antigorite (Atg) + calcite vein. (**h**) Pseudomorph crosscut by a late antigorite + calcite vein with the chrysotile replaced by antigorite. (**i**) Late growth of antigorite and calcite veins.

The cathodoluminescence (CL) of diopside results in emission of blue, yellowish green and blackish green radiation (Fig. 4). Most relict diopside has an obviously blue luminescence; less common are nonluminescent samples or those emitting blackish green radiation (dark) (Fig. 4a–c, g), while the margin shows green or yellowish luminescence. The calcite shows orange luminescence. Two-stage alteration processes can be recognized in serpentinized diopsidite. (1) Stage one involves formation of chrysotile + calcite by in situ alteration of diopside along grain boundaries or cleavages (Figs. 3d, e, 4a-d). The residual diopside is surrounded by nonluminescent chrysotile and calcite exhibiting orange luminescence (Fig. 4b–d). The minerals chrysotile and calcite can completely replace diopside to form pseudomorphic textures as well as network textures (Figs. 3f, 4e, f). (2) Stage two involves formation of antigorite + calcite, which occur as small veins (0.3–5 mm wide). The thin veins cut through the serpentinized diopsides and pseudomorphs (Figs. 3d, 4 g, h). The antigorite comprises fine-grained aggregates or coarse fibro-lamellar grains with interlocking and interpenetrating textures (Figs. 3g, 4i). The calcite is mainly present as coarse anhedral grains with twin lamellae (Fig. 3g).

### Compositions and submicrostructures of serpentinized diopsidites

**Figure 5:**
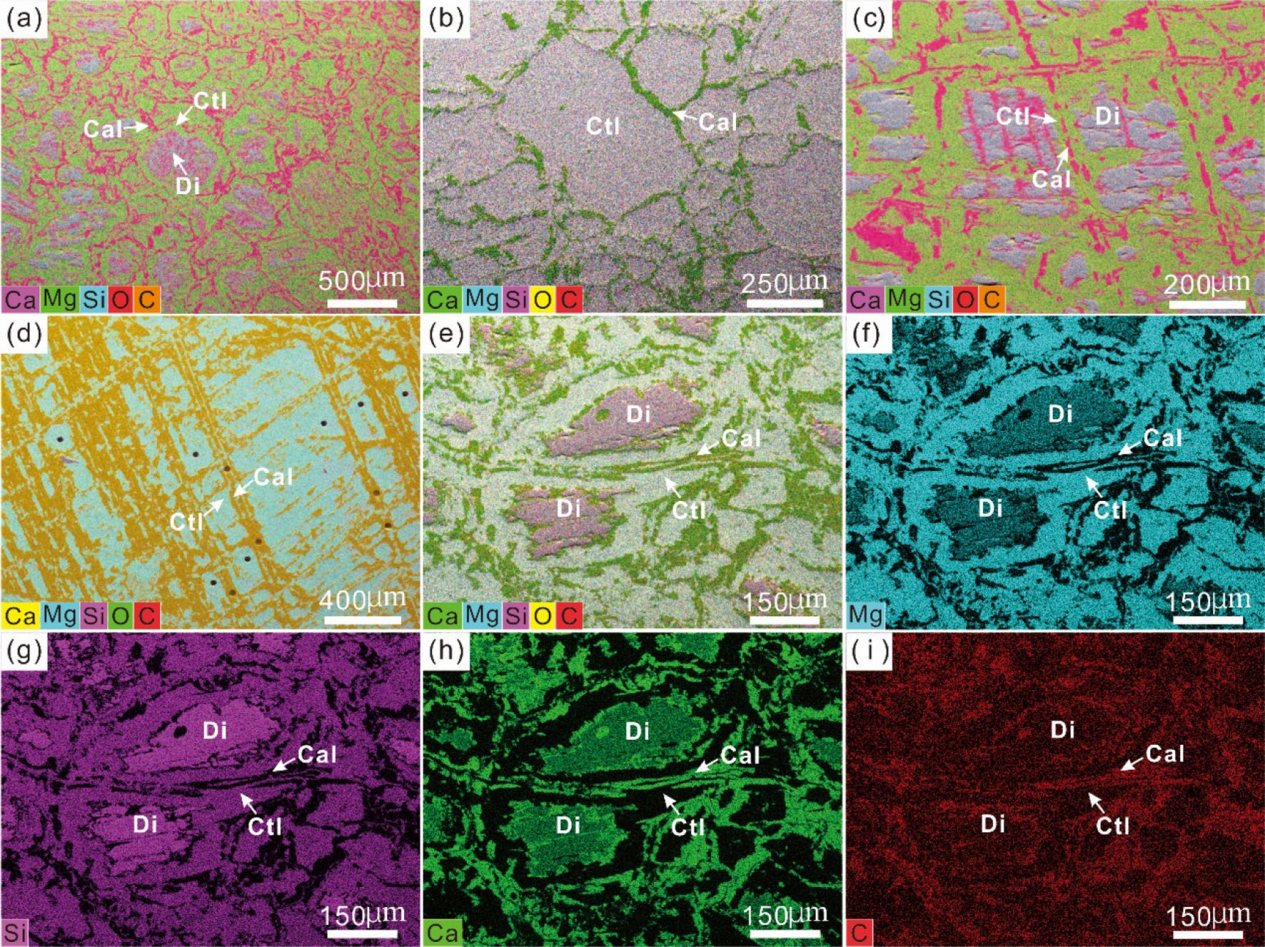
Composite element map images of serpentinized diopsidite in the Yushishan area. Composite element images are made by combining carbon (C) images with superimposed Mg, Ca, O, and Si images. (**a**) Serpentinization proceeded along the grain boundaries of diopsides. (**b**) Complete serpentinization of diopside forming pseudomorphs of chrysotile + calcite, without cleavage. (**c**) Serpentinization proceeded along the cleavages of diopsides. (**d**) Complete serpentinization of diopside forming pseudomorphs of chrysotile + calcite with cleavages. (**e–i**) Composite element image and Mg, Si, Ca, and C images.

Compositional mapping further shows the detailed alteration characteristics of diopside in the serpentinized diopsidites (Fig. 5). The grain boundaries of relict diopside grains were replaced by chrysotile + calcite that contain high concentrations of Mg and Ca, respectively (Fig. 5a). The completed serpentinization of diopside shows an obvious pseudomorph texture and no cleavages (Fig. 5b). Where the diopside was altered by chrysotile + calcite along the cleavages (Fig. 5c), it also formed pseudomorphs composed of chrysotile + calcite (Fig. 5d). The composition is consistent with the CL and microstructural data (Figs. 3 and 4). Furthermore, during alteration of diopside, the elements were redistributed to form chrysotile and calcite (Fig. 5e). Diopside has lower Mg but higher Si content than chrysotile (Fig. 5f, g). In contrast, the calcium in diopside was released and combined with carbon to form calcite (Fig. 5h, i).

**Figure 6:**
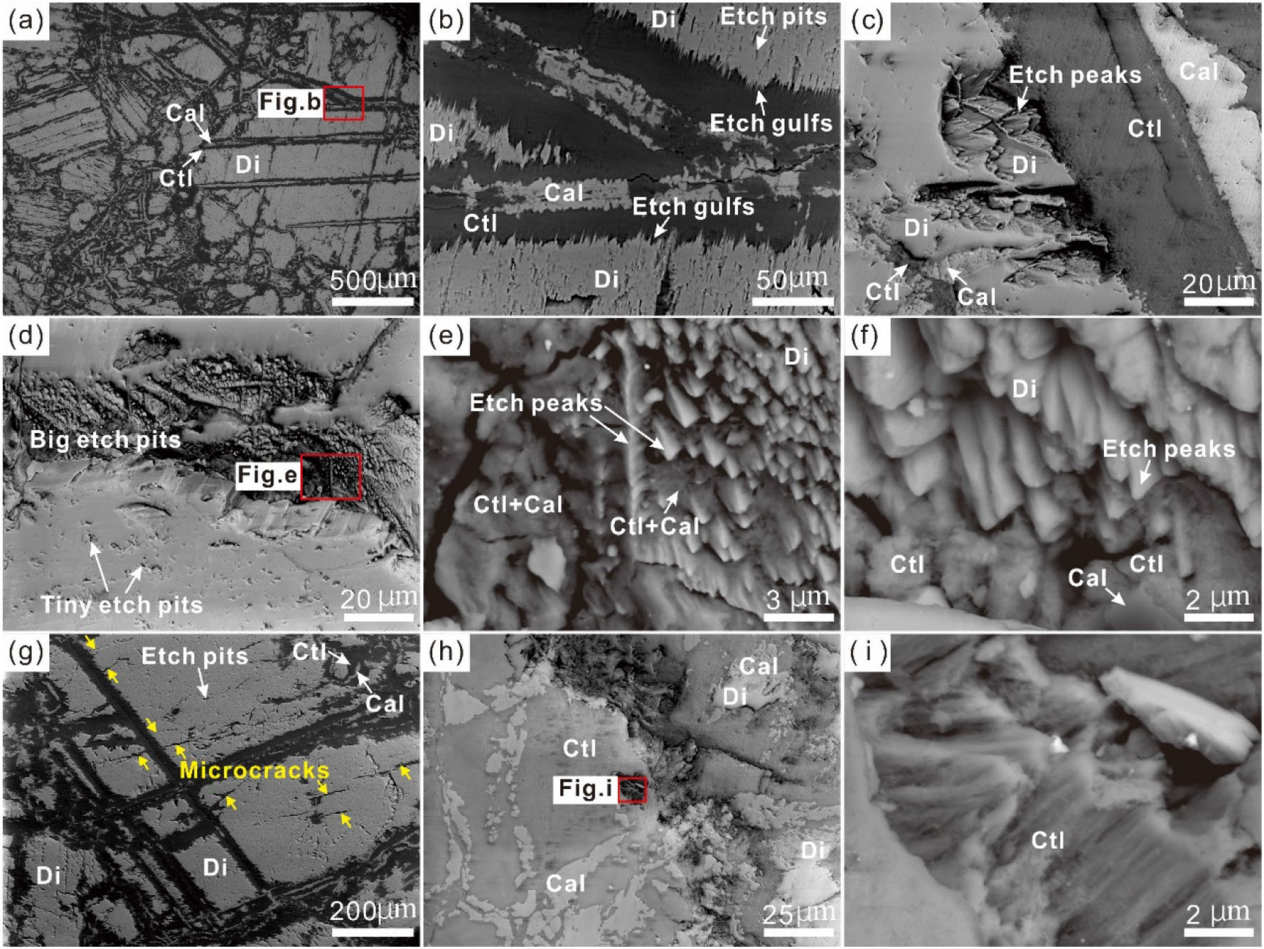
Backscattered electron (BSE) images of serpentinized diopsidite from the Yushishan area. (**a–c**) Many etch pits, etch gulfs, etc. peaks on diopside. (**d–f**) Conical-shaped and sawtooth-shaped etch peaks observed on diopside. Note the calcite and chrysotile around etch peaks. (**g**) Microcracks (yellow arrows) on diopside. (**h-i**) Fibrous chrysotile in pseudomorph.

The scanning electron microscopy backscatter (SEM-BSE) images show that many etch pits developed within the corroded boundaries or surfaces of the relict diopside grains (Fig. 6a, b). The minerals calcite and chrysotile also occur within the etch pits (Fig. 6c). The diopside presents wedge-shaped cross-sections of etch pits (i.e., etch gulfs) at the grain boundaries and cleavages (Fig. 6b, c). Within some large etch pits, the etch peaks have conical or sawtooth-shaped features (Fig. 6d–f). Rare microcracks can be observed within diopsides (Fig. 6g). The chrysotile in the pseudomorph exhibits a fibrous morphology texture (Fig. 6h, i).

### Bulk rock chemistry

The results of bulk rock major and trace element analyses from fresh and serpentinized diopsidites are presented in Supplementary Note 1. The fresh diopsidites have low Mg# (= 100 Mg/(Mg + Fe^2+^)) values (90.65–91.84, where only FeO measured by direct experiments is considered Fe^2+^, Fe_2_O_3_ is not included) and high SiO_2_ contents of 53.92–54.41 wt% and TFe_2_O_3_ contents of 1.36–1.62 wt%. They show low loss of ignition (LOI) of 1.18–2.10 wt%, indicating little alteration. In contrast, the serpentinized diopsidites have a very high LOI of 26.16 wt% due to the abundant formation of serpentine and calcite during alteration. It also has higher Mg# values (97.67) and MgO contents (23.77 wt%), with lower SiO_2_ contents of 34.40 wt%, Al_2_O_3_ contents of 0.02 wt%, and TFe_2_O_3_ contents of 0.66 wt%. All samples of diopsidites show very similar amounts of CaO (24.23–25.05 wt%), TiO_2_ (0.004–0.008 wt%), MnO (0.02–0.06 wt%), Na_2_O (0.03–0.07 wt%), K_2_O (0.008–0.012 wt%), and P_2_O_5_ (0.004–0.008 wt%).

**Figure 7:**
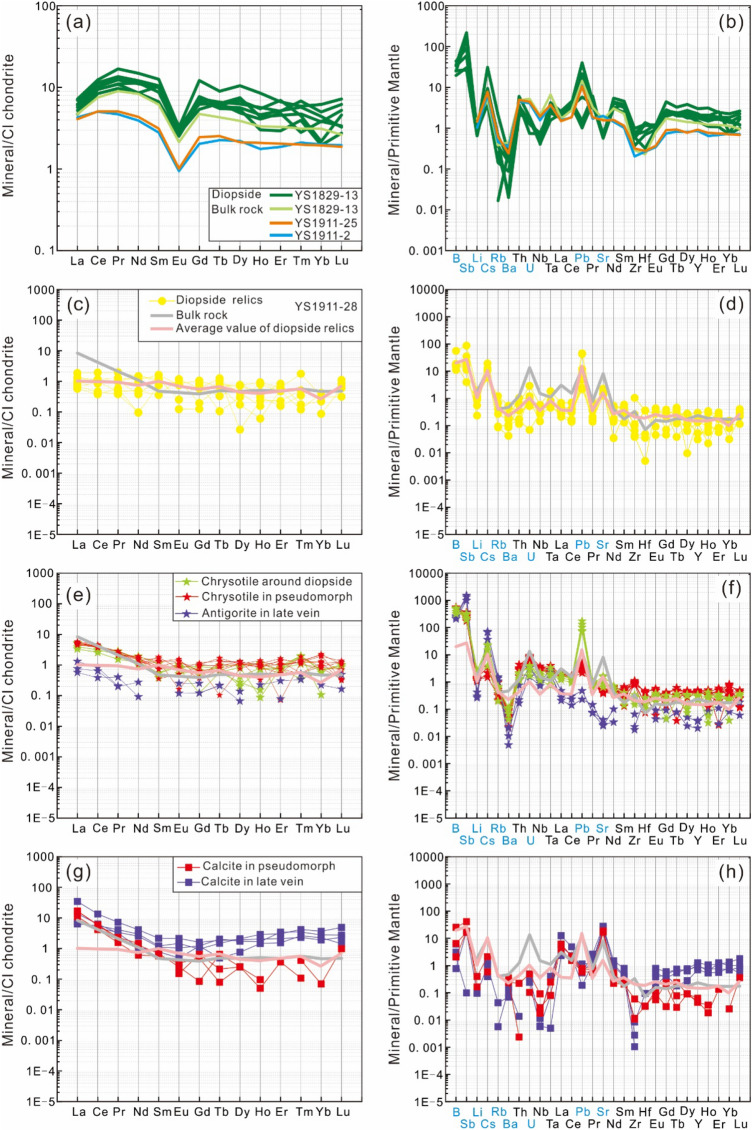
Chondrite- and primitive mantle-normalized diagrams. (**a, b**) Fresh diopsidites and diopsides; (**c, d**) diopside relics; (**e, f**) serpentines after diopsides and in late veins; (**g, h**) calcites after diopsides and in late veins. The (**a-c-e–g**) and (**b-d-f–h**) patterns are chondrite- and primitive mantle-normalized, respectively; normalizing values are from McDonough and Sun^47^. Blue fonts indicate fluid-mobile elements (FMEs). Grey and pink lines represent the values for the studied serpentinized diopsidite and the average value for diopside relics, respectively.

The normalized rare earth element (REE) and trace element patterns for the fresh diopsidite and serpentinized diopsidite are presented in Fig. 7. The fresh diopsidite features slight enrichment of light REEs (LREEs) relative to heavy REEs (HREEs) (1.51 < La_N_/Yb_N_ < 2.15; Fig. 7a) and strongly negative Eu anomalies ((Eu/Eu*) = 0.36–0.39). All samples of fresh diopsidites are significantly enriched in Pb ((Pb/Ce)_PM_ = 5.20–6.01; PM values from McDonough and Sun^47^). They show obvious depletions of Rb ((Rb/Th)_PM_ = 0.08–0.13), Ba ((Ba/Th)_PM_ = 0.05–0.09) and Zr and Hf ((Zr/Ce)_PM_ = 0.11–0.17; (Hf/Ce)_PM_ = 0.08–0.15; Fig. 7b).

The serpentinized diopsidite is characterized by flat HREE segments and strongly enriched LREE fractions (Yb_N_ = 0.47; La_N_/Yb_N_ = 17.87; La_N_/Sm_N_ = 17.60) (Fig. 7c). It shows HREE depletion compared to the fresh diopsidite and does not exhibit Eu anomalies ((Eu/Eu*) = 0.99). It shows obvious enrichments in Pb ((Pb/Ce)_PM_ = 8.28) that are similar to those of the fresh diopsidites (Fig. 7d). The serpentinized diopsidite is distinguished from the fresh diopsidites by its enriched Cs ((Cs/Th)_PM_ = 7.87), U ((U/Th)_PM_ = 10.93), and Sr ((Sr/Ce)_PM_ = 5.30) contents.

### Mineral chemistry

**Figure 8:**
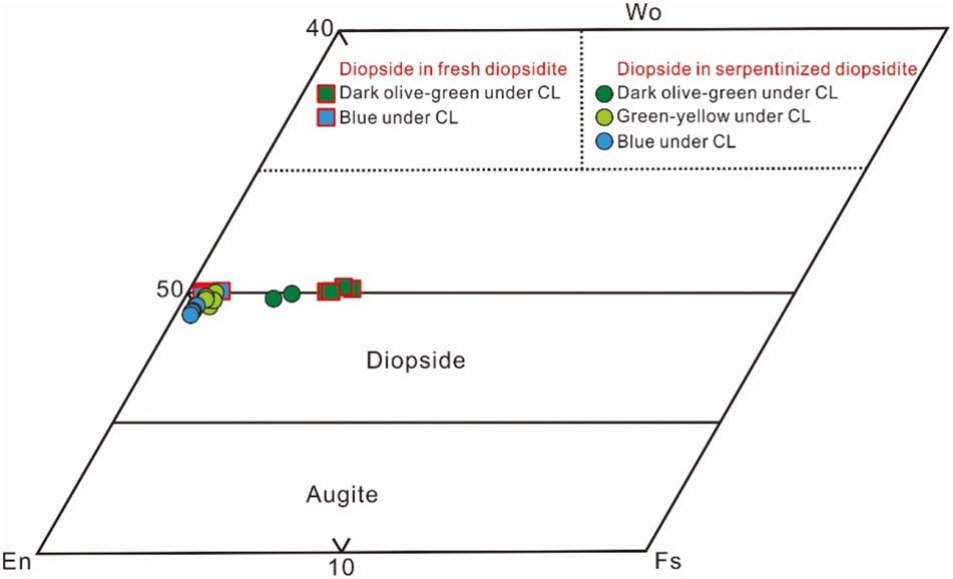
Mineral compositions of the pyroxenes in the Yushishan diopsidites. The CaSiO_3_(Wo)–MgSiO_3_(En)–FeSiO_3_(Fs) diagram (modified from Morimoto et al.^48^) shows that all pyroxenes are diopsides.

The main mineral compositions of diopside, serpentine and calcite were analysed by electron microprobe. The compositions of pyroxene in the fresh diopsidites as well as serpentinized diopsidite plot within the same field of diopside^48^ (Fig. 8). They are characterized by very high Mg# values (88.17–99.89, with an average of 97.62, where all Fe is considered Fe^2+^) and CaO contents (25.26–26.27 wt%) and low Al_2_O_3_ contents (0.02–0.10 wt%) and Cr_2_O_3_ contents (< 0.03 wt%). However, the diopsides that present a multi-coloured emission via CL have different contents of FeO_T_ and MgO. Blue and greenish yellow luminescent diopsides have lower FeO_T_ (0.04–0.81 wt%) and higher MgO contents (18.05–18.80 wt%). In contrast, blackish green luminescent diopsides show higher FeO_T_ contents (1.68–3.79 wt%) and lower MgO contents (15.84–17.66 wt%) (see Supplementary Note 2).

In particular, blackish green luminescent diopsides in the fresh diopsidite have higher REE abundances (∑REE = 16–28 ppm) and upwards convex chondrite-normalized REE patterns with strongly negative Eu anomalies (Eu/Eu* = 0.26–0.42; Fig. 7a). These diopsides are high in B, Sb, and Cs and low in Li, Rb, Ba, Sr, and HFSEs and show various Pb concentrations (Fig. 7b). Their chondrite-normalized REE patterns and primitive mantle-normalized trace element distribution patterns are similar to those of the bulk rock (Fig. 7a, b). In contrast, the diopsides of the serpentinized diopsidite are distinguished by lower REE abundances (∑REE = 0.54–3.26 ppm) and display relatively flat chondrite-normalized REE patterns with variable Eu anomalies (Eu/Eu* = 0.41–11.44; Fig. 7c). It is worth noting that the bulk rock of serpentinized diopsidite has an LREE-enriched (light rare earth element) pattern relative to that of the diopsides. The diopsides show particular enrichment in B, Sb, Cs, Pb, Sr and variable U concentrations (Fig. 7d). Overall, the diopside and bulk-rock diopsidite minerals both have very similar patterns of chondrite-normalized REEs and primitive mantle-normalized trace element distributions, except for the light REEs, U and Sr (Fig. 7c, d).

All serpentines (chrysotile and antigorite) are Mg-rich in serpentinized diopsidite, with Mg# of 97.62–99.27 (where all Fe is considered Fe^2+^; see Supplementary Note 2) and contain low CaO (< 0.3 wt%), NiO (< 0.04 wt%) and Cr_2_O_3_ (< 0.02 wt%) contents. Compared to antigorite, chrysotile has a higher Mg# of ~ 99 and lower contents of Al_2_O_3_ (0.03–0.08 wt%) and FeO_T_ (0.53–0.87 wt%). The major element concentrations of chrysotiles that formed either around relictic diopside or in pseudomorphs do not show obvious differences. In contrast, antigorite is usually characterized by lower Mg# values of 97–98 and higher Al_2_O_3_ and FeO_T_ contents (0.15–0.82 wt% and 1.24–2.29 wt%, respectively). Compared with chrysotile, antigorite contains lower REE contents. The chondrite-normalized REE patterns of serpentines match well that of the bulk rock, with strongly enriched LREEs (2.11 < La_N_/Yb_N_ < 36.88) and variable Eu anomalies (Eu/Eu* = 0.14–2.05; Fig. 7e), although serpentine has different amounts of REEs. Generally, primitive mantle-normalized trace element distribution patterns of serpentines resemble those of primary minerals (diopsides), except for the enrichments of LREEs and fluid-mobile elements (FMEs) (Fig. 7f). The serpentines are enriched in B (63–168 ppm) and Sb (0.95–8.34 ppm) and depleted in Ba (0.03–0.74 ppm) among fluid-mobile elements (FMEs). Additionally, the serpentines show somewhat different trace element compositions. In comparison with the chrysotile in pseudomorphs and antigorite, the chrysotile around diopside contains the highest Pb (10.67–25.85 ppm) and Sr (12.81–33.34 ppm) concentrations, while the chrysotile in pseudomorphs has the highest U (0.10–0.19 ppm) and Zr (8.09–11.7 ppm) concentrations but the lowest Cs (< 0.17 ppm) concentrations. Compared to chrysotile, antigorite displays lower trace element compositions, except for higher Sb (5.41–8.34 ppm), Cs (0.76–1.49 ppm) and Rb (0.64–0.91 ppm) concentrations (Supplementary Note 3; Fig. 7f).

The major element components of calcites in the serpentinized diopsidite show that all calcites contain low FeO_T_ (< 0.07 wt%), SrO (< 0.12 wt%) and MgO (0.23–0.60 wt%, except one ~ 2.05 wt%) contents (Supplementary Note 2). Due to the limited sizes of beam spots and small calcite grains, only the calcites in pseudomorphs and late veins were selected for analyses of trace element compositions. As with the serpentines, the calcites have LREE-enriched patterns (2.62 < La_N_/Yb_N_ < 157.76) and variable Eu anomalies (Eu/Eu* = 0.47–1.33; Fig. 7g). In particular, calcites in pseudomorphs contain lower HREE (heavy REE) contents than calcites in the late vein. When normalized to primitive mantle values, the trace element patterns of the calcites show positive anomalies in Sr, whereas Pb, Th and HFSEs (high field strength elements; e.g., Nb, Ta, Zr) are depleted (Fig. 7h). Compared to the trace element concentrations of serpentines, calcites have higher LREEs (especially La and Ce) and Sr levels, as well as lower levels of HFSEs (especially Nb and Ta) and some FMEs (e.g., B, Sb and U).

## Discussion

### Formation of fresh diopsidite

The diopsidite has been recognized as Ca-rich lithology, although its genesis is controversial (e.g., Ca-metasomatism^49^). The diopside-rich rocks are generally accepted to have magmatic or metamorphic origins^50^ and may be hydrothermal products of ultramafic–mafic rocks^51–53^. The magmatic-origin diopsidite crystallizes by partial melting of the enriched lithospheric mantle^54^, which is usually associated with the formation of alkaline magmatic rocks (e.g., syenite). It consists of diopside, biotite, magnetite and apatite. The hydrothermal-origin diopsidite generally forms network-like dikes within the surrounding altered peridotite or gabbro^52^. The main minerals are diopside, olivine, serpentine, chromite, magnetite, anorthite, garnet and chlorite^51,52^. In this study, the fresh diopside-rich diopsidites in the Yushishan area do not present the characteristics of magmatic or hydrothermal fluid reactions of ultramafic–mafic rocks. The studied diopsidites mainly comprise diopside ± tremolite ± phlogopite ± apatite ± zircon and occur together with serpentinized marble, garnet-bearing gneiss and amphibolite. In particular, the diopsidites are included in the serpentinized marbles (Fig. 2). Therefore, the diopsidites in the Yushishan area are more likely the decarbonation products of siliceous marble formed by contact progressive metamorphism^50,55–57^ or ultrahigh/high-pressure metamorphism^58,59^, although the metamorphism condition is not yet qualified. These observations that diopsidite directly contacted serpentinized marbles (Fig. 2e) and that diopside grains have dolomite inclusions (Fig. 3c) support the interpretation of carbonates decarbonize and react with silicate to form diopside.

### Coupled carbonation and serpentinization of diopsidite

It is interesting that carbonation and serpentinization of serpentinized diopsidite occurred simultaneously, which is supported by mineralogical and microstructural characteristics (Fig. 3). The pervasive occurrence of calcite as well as serpentine are seen for the same sample of serpentinized diopsidite (Figs. 3, 4 and 5). In particular, most diopsides were completely altered by calcite and chrysotile to form pseudomorphic textures (Figs. 3, 4e, f and 5b, d), which shows that carbonation was coupled to the serpentinization reaction. The diopsides in the serpentinized diopsidite provide evidence of dissolution and precipitation at the mineral-fluid interface, and every relict diopside grain developed many etch pits (Fig. 6b). The wedge-shaped cross-sections (i.e., etch gulfs) of the etch pits are corroded at the grain boundaries between diopside and chrysotile (Fig. 6b, c). Calcite and chrysotile precipitated around the etch peaks after the diopside dissolved (Fig. 6e, f). The formation and features of the etch pits display remarkable similarities with the results for olivine^60,61^ and pyroxene^15,27,40,62^, which underwent selective etching by aqueous dissolution on their surfaces during mineral reactions and alteration. In particular, a pervasive pseudomorph texture is present in the serpentinized diopsidite (Figs. 3, 4, 5 and 6), indicating spatial coupling between dissolution and precipitation^63–66^.

Serpentinization of olivine and/or orthopyroxene is often accompanied by the occurrence of reaction-induced fracturing^67–70^. However, no pervasive fractures are identified in the serpentinized diopsidite studied here (Fig. 6), although a few microfractures can be observed. Indeed, the petrographic data show that diopside alteration occurred in the cleavages and grain boundaries and formed chrysotile + calcite (Figs. 3, 4, 5 and 6). This suggests that the cleavage and grain boundaries in diopside can facilitate coupling of carbonation and serpentinization. It is generally suggested that chrysotile and lizardite are stable under low-pressure and low-temperature conditions (0–300 °C, P < 1.0 GPa)^71^, although the formation conditions and mechanism for the antigorite are debatable^72^. Traditionally, antigorite has been considered a high-temperature and high-pressure serpentine phase^73^, but it can also form at low temperature ± pressure^72,74^. In this study, diopside was altered to form chrysotile, antigorite and calcite. The temperature and pressure conditions of the diopside conversion to chrysotile + calcite cannot be estimated accurately for the samples studied in the present situation.

### Element migration during diopsidite serpentinization and carbonation

There are controversial isochemical or nonisochemical processes proposed for serpentinization and carbonation reactions. In an isochemical process, the serpentinization and carbonation reactions exhibit no or very minor changes in the major element composition of the serpentinized peridotite compared to that of the protolith, except for H_2_O and CO_2_^34,75,76^. In contrast, nonisochemical processes lead to larger changes in major element chemistry, such as Si enrichment^77,78^, Mg loss or enrichment^77,79,80^ and Ca loss or enrichment^29,32,81,82^. The studied samples of serpentinized diopsidites have higher MgO/SiO_2_ ratios than the fresh diopsidites (Supplementary Note 1). Moreover, the serpentinized peridotite shows higher LOI and lower SiO_2_ concentrations but has similar amounts of CaO compared to the fresh diopsidites (where the major element contents are described as anhydrous compositions). Certainly, SiO_2_ is lost during the serpentinization and carbonation of diopsidite, although it is unclear where the SiO_2_ went. Furthermore, the serpentinized diopsidite has a higher LOI because it contains more serpentines and calcite than all of the fresh diopsidites (Supplementary Note 1). Because the tectonic and metamorphic settings of the Yushishan area remain unclear, the sources of H_2_O and CO_2_ are uncertain. However, the serpentinization and carbonation of diopside could follow the simplified reaction:

1 $$ {\text{3 CaMgSi}}_{{2}} {\text{O}}_{{6}} + {\text{ 2 H}}_{{2}} {\text{O }} + {\text{ 3 CO}}_{{2}} = {\text{ Mg}}_{{3}} {\text{Si}}_{{2}} {\text{O}}_{{5}} \left( {{\text{OH}}} \right)_{{4}} + {\text{ 4 SiO}}_{{{2}({\text{aq}})}} + {\text{ 3 CaCO}}_{{3}} $$

**Figure 9:**
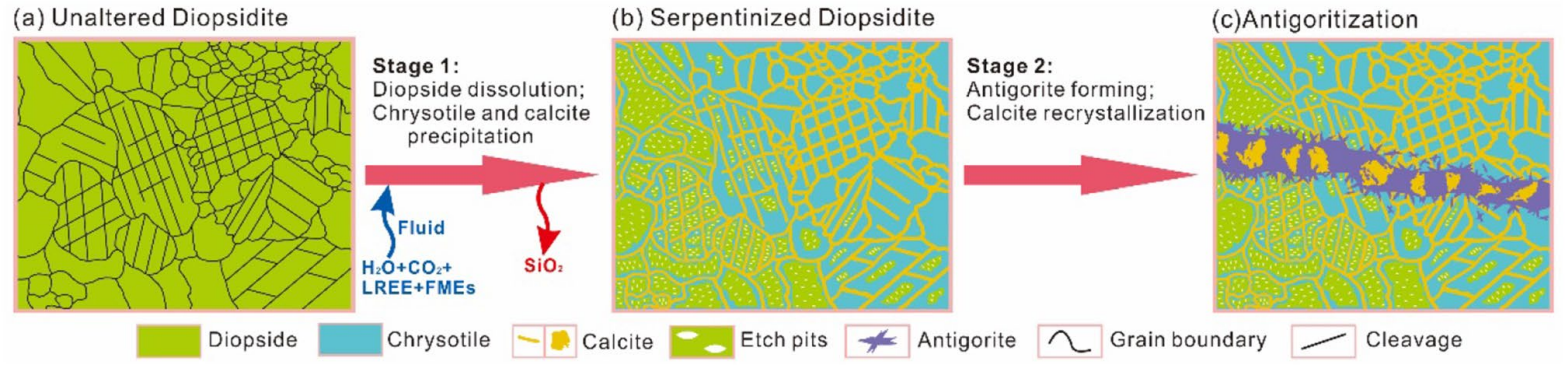
Sketch representing the formation processes of the studied serpentinized diopsidite. (**a**) Fresh diopsidite with abundant cleavages. (**b**) CO_2_-rich fluids flow along the grain boundaries and cleavages of diopsides. Diopsides dissolve to form abundant etch structures (e.g., etch pits); simultaneously, calcite and chrysotile nucleate and grow around the etch peaks. Then, diopsides are completely altered by calcite and chrysotile and form pseudomorphic textures. Moreover, this process allows the altered diopsidite to acquire additional H_2_O, CO_2_, LREEs, and FMEs but lose SiO_2_. (**c**) Serpentinized diopsidite further undergoes antigoritization that results in the formation of antigorite and coarse-grained calcite.

The LREE enrichments, as shown by the high LREE/HREE ratios in serpentinized rocks, could be the result before serpentinization^83,84^. Moreover, during serpentinization, the changes in the REE budget can be moderated^76^, and LREEs can be added obviously^85,86^. In the studied serpentinized diopsidite samples, the hydrothermal processes of serpentinized diopsidite are recorded in both chrysotile and calcite that display enrichment in LREEs with flat HREE segments (Fig. 7e, g). In contrast, the residual diopsides in the serpentinized diopsidite display relatively flat chondrite-normalized REE patterns (Fig. 7c). Compared to the fresh diopsidites, although the abundance of HREEs in the serpentinized diopsidite is lower, the LREEs (notably La) are more enriched than the HREEs. Therefore, the processes of both serpentinization and carbonation lead to LREE enrichment of the bulk rock. Previous studies of serpentinized peridotites suggested that serpentinization can prompt element migration and redistribution of fluid-mobile elements (FMEs), such as Cs, U, Sr, B, Li, Sb and Rb^76,87^. In our samples, the serpentinized diopsidite has higher contents of fluid-mobile elements (FMEs; U, S, B, Sb) than the primary phase fresh diopsidites (Fig. 7). The elements U and Sr are enriched in chrysotile and calcite, respectively (Fig. 7f, h), and B and Sb are enriched in serpentine (Fig. 7d, f, h), indicating that enrichment was the result of serpentinization and carbonation. Hence, the results reveal two-stage alteration processes for serpentinized diopsidite: (1) CO_2_-bearing fluids flowed along the cleavage planes and grain boundaries of diopsides. The diopsides dissolved and chrysotiles and calcites precipitated via a coupled dissolution–precipitation mechanism. The serpentinized diopsidite added CO_2_, H_2_O, LREE, and FMEs and lost SiO_2_. (2) Further antigoritization resulted in the formation of antigorite veins and recrystallized coarse calcite (Fig. 9).

### Implications for carbon capture and storage

Carbon capture and storage (CCS) processes reduce or eliminate the net flux of carbon dioxide into the atmosphere to avoid dangerous climate change^8^. Mineral carbonation is considered a safe and permanent way to store CO_2_^1,2^. In the last 30 years, based on natural samples and experimental studies, some preponderant rocks, such as basalt, peridotite and serpentinite, have been studied for permanent CO_2_ storage by mineral carbonation^3–6,31,35,88^. Currently, there are two large-scale field projects (i.e., the Icelandic CarbFix project and the American Wallula project) underway to study basalt formations for in situ mineral carbonation^89,90^. Diopside (CaMgSi_2_O_6_) is a suitable mineral for CO_2_ sequestration due to its richness in Mg and Ca. In natural outcrops, diopside-rich rocks with different origins (e.g., diopsidite) have been found in various geological settings, such as in ophiolites and skarns. It has been proposed that the diopsidites from the Oman ophiolite were formed by Ca enrichment in serpentinite and induced by the circulation of high-temperature hydrothermal fluids^51^. Clinopyroxene (diopside in particular) is normally a major constituent in skarns^55,91^ and can be formed by contact metamorphism at global continental arcs that are long-term storage sites for sedimentary carbonates^50^. The diopsidite can be the product of uppermost mantle harzburgite or lowermost crustal gabbro reactions with hydrothermal fluids^52^. The diopsidite can also crystallize by partial melting of the enriched lithospheric mantle^54^. The magmatic diopsidite can be exposed over a large area constituting several square kilometres^54^, although the global amount of diopsidite cannot be determined. A few experimental studies have probed the carbonation process of diopside^13,14^. The abundant calcite forms during carbonation coupled with serpentinization, as presented here in the study of serpentinized diopsidite (Fig. 3), under natural conditions. Therefore, diopside-rich rocks show promise for CO_2_ storage via coupled dissolution and precipitation processes. The CCS or carbon cycle significance of exposed diopside-rich rocks should not be neglected, although further work is needed to estimate how much CO_2_ can be stored in diopside-rich rocks in a global context.

## Methods

### Petrography analysis

Optical cathodoluminescence (CL) imaging was carried out using a Zeiss A1 microscope coupled with a Beacon Innovation International Inc. (BII) CLF-2CL system at the Experimental Center, School of Earth Sciences, China University of Geosciences (Wuhan). The CL system was operated at an accelerating voltage of ~15 kV and a current of ~280 mA.

Raman microspectroscopy was conducted at the State Key Laboratory of Biogeology and Environmental Geology, China University of Geosciences (Wuhan) using a wITec ɑ 300 Confocal Raman system coupled with a Peltier-cooled EMCCD detector. Laser excitation at 532 nm with an output power between 3 and 10 mW was used. Spectra were obtained using 100 x (N.A.= 0.9) magnification objectives with a 50 μm diameter optic fibre. A 600 g/mm grating was used, yielding a spectral resolution of ~4 cm^-1^. Raman spectra were collected at confocal depths of at least 1 μm below the thin section surface. WITec Project Five 5.1 Plus software was used to process all Raman spectra. The spectra were processed with a background subtraction polynomial fit, typically on the order of 4–6. The Raman spectra of serpentine species exhibit peaks in the OH stretching range (3550–3800 cm^−1^).

Field-emission scanning electron microscopy (FI-SEM) studies were performed at the Experimental Center, School of Earth Sciences, China University of Geosciences (Wuhan) using a new Sigma 300VP FEG-SEM field emission scanning electron microscope with an energy dispersive spectrometer (EDS) detector for detailed submicroscopic microstructural analyses. Scanning electron microscopy was used to obtain backscatter diffraction images with a spot size of 6.0 mm. The beam current and accelerating voltage were set at 15 nA and 20 kV, respectively; the working distance was ~12 mm. The specified dwell time was > 20 min.

### Geochemistry of bulk rocks

Whole-rock compositions were analysed at Wuhan Sample Solution Analytical Technology Co., Ltd., Wuhan. Whole-rock major element analyses were performed by the melting method. The flux was a mixture of lithium tetraborate, lithium metaborate and lithium fluoride (45:10:5), and ammonium nitrate and lithium bromide were used as oxidants and release agents, respectively. The melting temperature was 1050 ℃, and the melting time was 15 min. A Zsx Primus II wavelength dispersive X-ray fluorescence spectrometer (XRF) produced by RIGAKU, Japan, was used for analyses of the major elements in the whole rock. The X-ray tube was a 4.0 kW end window Rh target. The test conditions involved a voltage of 50 kV and a current of 60 mA. All major element analysis lines are kα. The standard curve used the national standard material, rock standard sample GBW07101-14. The data were corrected by the theoretical α coefficient method. Relative standard deviations (RSDs) were less than 2%.

Whole-rock trace element analyses were conducted with an Agilent 7700e ICP‒MS at Wuhan Sample Solution Analytical Technology Co., Ltd., Wuhan, China. The detailed sample-digestion procedure was as follows: (1) Sample powder (200 mesh) was placed in an oven at 105 ℃ and dried for 12 h; (2) 50 mg of the sample powder was accurately weighed and placed in a Teflon bomb; (3) 1 ml of HNO_3_ and 1 ml of HF were slowly added into the Teflon bomb; (4) the Teflon bomb was put in a stainless steel pressure jacket and heated to 190 ℃ in an oven for > 24 h; (5) After cooling, the Teflon bomb was opened and placed on a hotplate at 140 ℃ and evaporated to incipient dryness, and then 1 ml of HNO_3_ was added and evaporated to dryness again; (6) 1 ml of HNO_3_, 1 ml of MQ water and 1 ml of a 1 ppm internal standard solution were added, and the Teflon bomb was resealed and placed in the oven at 190 ℃ for > 12 h; (7) the final solution was transferred to a polyethylene bottle and diluted to 100 g by the addition of 2% HNO_3_. Rock standards BHVO-2, GSR-1, and GSR-3 were used as external standards. The accuracy was generally > 10%. The results are presented in Supplementary Note 1.

### Mineral chemistry

Major elements were identified with a JEOL JXA-8230 electron microprobe at Wuhan Sample Solution Analytical Technology Co., Ltd., Wuhan, China. The operating conditions were as follows: 15 kV accelerating voltage, 20 nA cup current, 1 μm (for diopside) or 3 μm (for serpentine and calcite) beam diameter. A series of natural minerals were used as standards for calibration. Raw data were corrected using a ZAF algorithm (where Z = element atom number, A = X-ray absorption, F = X-ray fluorescence). The results are presented in Supplementary Note 2.

Trace element analyses of minerals were conducted by LA-ICP‒MS at Wuhan Sample Solution Analytical Technology Co., Ltd., Wuhan, China. Laser sampling was performed using a GeoLasPro laser ablation system that consisted of a COMPexPro 102 ArF excimer laser (wavelength of 193 nm and maximum energy of 200 mJ) and a MicroLas optical system. An Agilent 7700e ICP‒MS instrument was used to acquire ion-signal intensities. Helium was used as the carrier gas. Argon was used as the make-up gas and mixed with the carrier gas via a T-connector before entering the ICP. The spot size and frequency of the laser were set to 44 µm and 5 Hz, respectively, in this study. The trace element compositions of minerals were calibrated against various reference materials (BHVO-2G, BCR-2G and BIR-1G). Each analysis incorporated a background acquisition of approximately 20–30 s followed by 50 s of data acquisition from the sample. The Excel-based software ICPMSDataCal was used to perform off-line selection and integration of background and analysed signals, time-drift correction and quantitative calibration for trace element analysis. The results are presented in Supplementary Note 3.

## Supplementary Information

Supplementary Information 1.

Supplementary Information 2.

Supplementary Information 3.

Supplementary Information 4.

## Data Availability

All data used in this contribution is available in the main text, supplementary files, or in the cited papers. Any questions regarding the data should be directed to the communicating authors.
