# Supplementary material for: Carbonation and serpentinization of diopsidite in the Altun Mountains, NW China
Source: Sci Rep. 2022 Dec 9;12:21361. doi: 10.1038/s41598-022-25612-5 (PMC9734160; doi:10.1038/s41598-022-25612-5)
Supplement: Supplementary file 1 — Supplementary Information 1. [file 41598_2022_25612_MOESM1_ESM.pdf]

Description of Additional Supplementary Files

File Name: Supplementary Note 1
Description: Bulk-rock major (wt.%) and trace element (ppm) analyses for Yushishan diopsidites.

File Name: Supplementary Note 2
Description: EPMA analyses of diopsides, serpentines and calcites from Yushishan diopsidites.

File Name: Supplementary Note 3
Description: LA-ICPMS analyses of diopsides, serpentines and calcites from Yushishan diopsidites.
